# Negative Results in Neonatal Trials: Clinical Lessons and Future Directions – A Narrative Review

**DOI:** 10.1159/000549325

**Published:** 2025-11-04

**Authors:** Ashraf Gad, Ammar Yasser Nofal, Leena Khalid Al-Qassem, Loay Alkamel

**Affiliations:** a Critical Care, Neonatal Intensive Care Unit, Women’s Wellness and Research Center, Hamad Medical Corporation, Doha, Qatar; b Weill Cornell Medicine-Qatar, Doha, Qatar; c Misr University for Science and Technology, Giza, Egypt; d College of Medicine, QU Health, Qatar University, Doha, Qatar; e Qatar National Library, Doha, Qatar

**Keywords:** Clinical trials, Neonatology, Negative results, Null hypothesis, Research, Neonatal intensive care unit

## Abstract

**Background:**

Clinical research involving neonates often presents unique ethical and practical challenges. These difficulties may lead to the early termination of clinical trials, particularly when negative or inconclusive results occur, contributing to publication bias. Failure to report such outcomes ultimately slows the advancement of knowledge and the development of evidence-based care in neonatology.

**Summary:**

This review examines the significance of negative trial results in neonatal clinical research, with particular emphasis on interventional studies, and addresses their reliability, challenges in interpretation, and implications for clinical practice. Insights from selected negative neonatal trials were used to assess the impact of unfavorable outcomes on neonatal care. Non-interventional studies were excluded from this review to maintain a focus on controlled clinical trials.

**Key Messages:**

Properly interpreted negative trials hold significant value in neonatal research. These studies help avoid unnecessary interventions, ensuring more efficient use of resources, and guide future research directions. Despite often being undervalued or overlooked, they remain fundamental to advancing evidence-based neonatal care. Enhanced reporting and interpretation of these findings could greatly benefit both clinical practice and research development in the neonatal population.

## Introduction

### Negative Results in Clinical Research

In clinical research, “negative results” refer to trials that fail to demonstrate statistically significant differences between treatment and control groups, support the null hypothesis (the assumption that there is no effect), or reveal an effect opposite to what was hypothesized [1, 2]. While often underreported, these results are crucial. Negative results challenge assumptions, expose methodological limitations, and help tailor future hypotheses to more specific or nuanced questions [3].

Negative trials are essential in refining hypotheses and reducing publication bias. Publication bias occurs when studies with positive outcomes are more likely to be published, cited, and incorporated into clinical guidelines [4]. Such bias distorts the scientific literature and can lead to overestimation of treatment efficacy. By allowing the scientific community to evaluate the full body of evidence, whether supportive and refutative, negative findings play a corrective role. This ensures that interventions are based on balanced and transparent data [5, 6].

### Evidence Gaps and Publication Bias in Neonatology

The challenges surrounding evidence are especially pronounced in neonatology, where interventions often precede robust data, and practice is shaped by tradition, small studies, or evolving consensus. Over 50% of neonatal systematic reviews are inconclusive [7]. This concern has been increasingly recognized as a global public health issue, as highlighted by the Lancet Commission on the Future of Neonatology [8]. The commission called for innovation, coordinated trial infrastructure, and collaborative investment to generate high-quality evidence in neonatal medicine.

Many neonatal and pediatric randomized controlled trials (RCTs) do not reach completion, with common barriers including slow or insufficient enrollment, logistical problems, and financial challenges. A cross-sectional review of pediatric RCTs revealed that 19% of trials were terminated prematurely [9], while 11% of trials were terminated prematurely in ClinicalTrials.gov entries [10], 9.2% in research related to pregnancy [11], and 18% of neonatal studies in the ANZCTR registry [12]. Early discontinuation of trials frequently leaves studies underpowered, thereby increasing the risk of false-negative outcomes.

Furthermore, neonatal RCTs with unfavorable findings are less likely to be published in high-impact journals compared to trials with favorable or statistically significant findings. This discrepancy in selective reporting can negatively impact the development of new research questions or care pathways by affecting the evidence base and jeopardizing the clinical utility of trial results [3]. In neonatology, where patient safety and long-term outcomes are paramount, recognizing and embracing negative results is not only a scientific imperative but also a clinical necessity.

The aim of this narrative review is to analyze the clinical and methodological significance of negative results in neonatal interventional trials, to discuss their reliability, and to highlight how such findings, when robustly designed and transparently reported, can inform practice and future research directions. Importantly, the influence of a trial depends less on whether its results are positive or negative, and more on its methodological rigor, statistical power, transparent registration, and use of clinically relevant outcome measures.

## Challenges in Interpreting Negative Trials

Negative trials can offer high-quality evidence to abandon ineffective or harmful interventions, but only when they are adequately powered, rigorously designed, and report complete outcome data. In neonatology, however, interpreting negative or null results is often challenging. Several methodological and practical issues increase the risk of false-negative findings (type II errors) or inconclusive conclusions.

A fundamental limitation in neonatal research is that many trials are statistically underpowered, rendering them unable to detect clinically meaningful effects. This issue is pervasive; for instance, in neonatal clinical trials, most outcomes lack robustness: a Cochrane review found 78% of outcomes have low/very low certainty [13], and a review of 262 trials (mainly preterm infants) reported up to 58% of outcomes as inconclusive [14].

The problem is exacerbated by incomplete outcome data. A specific review of 87 neonatal and perinatal trials demonstrated that incomplete outcome reporting was the norm, with only 11% of studies containing full data sets; this routinely leads to findings that are either inconclusive or lack the precision to guide practice [15].

Variability in outcome definitions complicates the interpretation of meta-analyses. For example, definitions of bronchopulmonary dysplasia (BPD) vary; some studies define BPD as oxygen dependence at 36 weeks postmenstrual age, while others use physiologic assessments. This lack of standardization limits the ability to accurately assess the effects of treatment.

Moreover, trials with wide confidence intervals around effect estimates are often misinterpreted as negative. However, a “negative” trial with confidence intervals that include clinically meaningful benefit, or harm cannot be interpreted as proving equivalence or absence of effect [16]. These ambiguous findings add to uncertainty rather than resolving clinical questions.

Further bias arises from post hoc subgroup analyses and selective outcome reporting. Exploratory analyses not prespecified in the protocol can inflate observed effects and distort interpretation. Protocol deviations also undermine the validity of trial findings. For example, in the APTS trial [17], 15% of infants did not receive the assigned intervention, and contamination – such as time spent above lower target oxygen saturations – was observed in the COT [18] and BOOST-II [19] trials, diluting potential harm and biasing results toward the null.

## Methods

This review was conducted as a narrative synthesis rather than a scoping or systematic review, since neonatal trials rarely label themselves as “negative” or “null” in titles, abstracts, or registries, making a reproducible eligibility strategy unfeasible. We purposively selected illustrative interventional trials that failed to confirm their primary hypothesis, chosen for clinical relevance, trial size, and impact on neonatal practice. Evidence was gathered from PubMed, Medline, Cochrane, and Google Scholar, as well as relevant reviews, commentaries, and editorials, without year restrictions. While observational studies and registries provide valuable impact on clinical practice, insights into safety signals and long-term outcomes, this review focused primarily on interventional trials, as they most directly influence clinical practice and guideline development.

## Classification and Case Studies of Key Negative Trials in Neonatology

Negative neonatal trials can be categorized based on why they were negative and their clinical implications (Table 1).

**Table 1.** Negative neonatal trials: classification, clinical implications, and lessons learned

Type of negative trial	Intervention	Trial (year)	Design	Primary outcome/objective	Key findings	Why negative?	Justification for classification	Clinical implications	Lessons learned
True negative	Erythropoietin (Epo) for neuroprotection	PENUT (2020) [20, 21]	Multicenter RCT (*n* = 941)	Death or severe NDI at 22–26 m	No difference in reducing severe NDI or MRI brain injury scores at term	Large, definitive RCT	Well-powered study with long-term follow-up, with a definite null result	Disproves Epo for neuroprotection	Even strong mechanistic rationale (neuroprotection) requires RCT validation 941 infants sufficient to rule out clinically meaningful benefit Highlights need for alternative neuroprotective strategies
True Negative	Probiotics (*B. breve*) for NEC	PIPS (2016) [22]	Multicenter RCT (*n* = 1,315)	bloodstream infection, or NEC ≥ Stage II between 72 h and 46 weeks PMA or death before discharge	No reduction in NEC/death	Adequately powered for primary outcome, null result	Well-designed trial with sufficient sample size	Suggested that not all probiotics are the same, need further studies with different strains and doses	Not all probiotic strains are equal - strain-specific effects matter Large sample (*n* = 1,315) still showed null effect Need for precision microbiome approaches
Harmful negative	Early inhaled budesonide for BPD prevention	NEUROSIS (2015) [23]	Multicenter RCT (*n* = 863)	Death or BPD at 36 weeks PMA	Reduced BPD (27.8% vs. 38.0%, RR 0.74, *p* = 0.004) but increased mortality trend (16.9% vs. 13.6%, RR 1.24, *p* = 0.17)	Mortality-BPD trade-off	Underpowered for mortality; unclear risk-benefit	Inhaled steroids may reduce BPD but require mortality safety studies	BPD reduction may come at mortality cost Inhaled steroids still have systemic effects Risk-benefit balance unfavorable in most cases
True negative	Early treatment with Ibuprofen for large PDA	Baby-OSCAR (2024) [24, 25]	Multicenter RCT (*n* = 653)	Death or mod-severe BPD at 18–22 months PMA	No significant difference between early ibuprofen treatment and placebo in primary outcome	Early ibuprofen treatment did not improve outcomes compared to placebo	Well-powered trial, but no significant benefit observed despite sufficient sample size	Ibuprofen may close PDA but does not improve clinical outcomes, potentially altering treatment protocols for EP infants	PDA closure does not mean improved clinical outcomes Largest PDA trial confirms futility of early treatment Challenges decades-old practice patterns
				NDI & respiratory outcomes at 2 years (corrected)					
False negative	Dopamine vs. placebo for hypotension in preterm infants	HIP trial (2021) [26]	Multicenter RCT (*n* = 58; planned *n* = 830)	Survival to 36 weeks postmenstrual age without severe brain injury	No significant difference between groups, however, more additional interventions were needed in the placebo group	Early termination due to recruitment challenges; small sample size limited statistical power	Despite trends suggesting potential differences, the study was underpowered to detect statistically significant effects	Questions the routine use of dopamine for mild hypotension in this population suggesting inotropes may not lead to worse outcomes	Highlighted recruitment challenges in neonatal trials 58 infants insufficient for conclusions Need for alternative trial designs
False negative	High-flow nasal cannula (HFNC) vs. CPAP for initial respiratory support in preterm infants	HIPSTER Trial (2016) [27]	Multicenter RCT (*n* = 564)	Non-inferiority trial, treatment failure within 72 h despite maximal respiratory support	HFNC had higher treatment failure vs. CPAP, but no significant difference in intubation or adverse events; early termination due to safety concerns. Suggests HFNC may still be a viable option	HFNC had a higher treatment failure rate than CPAP. However, early termination and underpowering suggest the results might not reflect HFNC's true potential	Underpowered due to early termination, limiting the ability to draw definitive conclusions	HFNC may be considered in selected cases or with modified protocols, but CPAP remains the preferred initial support modality	Simple interventions still need rigorous testing 564 infants showed clear null effect Ended/reduced popular off-label practice
True negative	Inhaled nitric oxide for preterms	EUNO (2010) [28]	Multicenter RCT (*n* = 800)	survival without BPD at 36 weeks	No benefit	Large RCT with clear null result	Large, well-designed confirmatory trial	iNO should not be used routinely for prevention of BPD in preterm infants	Confirmed NINOS findings in larger cohort (*n* = 800) Ended iNO as BPD prevention strategy Showed limits of physiologic rationale alone
Equivocal negative	Whole-body hypothermia for mild neonatal HIE	COMET (2024) [29]	Pilot multicenter RCT (*n* = 101)	Cerebral MRI biomarkers at 4–7 days postnatal age	No improvement in MRI biomarkers; hypothermia groups sicker at baseline	Underpowered, open label, surrogate endpoint, baseline imbalance	Pilot study with inconclusive efficacy but no clear harm	Larger RCTs needed for mild HIE	Mild HIE may require different neuroprotective approaches than moderate/severe HIE MRI biomarkers alone may not capture clinical benefits Highlights need for larger trials with clinical endpoints
True negative	Prophylactic oropharyngeal surfactant	POPART (2024) [30]	Multicenter RCT (*n* = 251)	Intubation within 120 h of birth	No difference in intubation rates between groups	Well-powered, rigorously conducted trial	Large, multicenter trial with a clear null result	Prophylactic oropharyngeal surfactant should not be routinely used	Simple interventions still need rigorous testing 251 infants showed clear null effect
False negative	Docosahexaenoic acid, choline, and uridine-5-monophosphate supplementation in infants at risk of neurodevelopmental impairment	DOLPHIN Trial (2018) [31]	RCT (*n* = 59)	Cognitive development at 24 months using Bayley-III CCS	No statistically significant difference in cognitive scores between intervention and control groups	Supplements did not significantly improve cognitive outcomes	The small sample size limited the ability to detect significant differences. Subsequent studies with larger cohorts have shown benefits of nutritional supplementation in similar populations	More research with larger sample size needed to explore supplementation to prevent neurodevelopmental delay	Underpowered (*n* = 59) despite good design Highlights danger of small negative trials
Opposite effect	Sildenafil for severe fetal growth restriction	Dutch STRIDER (post hoc 2022) [32]	Secondary analysis of RCT (*n* = 163 live births)	Pulmonary hemorrhage mechanisms in exposed neonates	Increased PH (RR 3.67, 95% CI 1.28–10.51, *p* = 0.02) Rebound vasoconstriction hypothesized	Paradoxical harm despite fetal vasodilation theory	Mechanistic plausibility reversed postnatally	Contraindicates sildenafil for severe FGR	Fetal vasodilation does not guarantee postnatal benefits Drugs may have paradoxical effects across developmental transitions Requires separate fetal vs. neonatal efficacy/safety trials
True negative	Indomethacin prophylaxis for IVH and PDA	TIPP (2001) [33]	Multicenter RCT (*n* = 1,202)	Composite of death, cerebral palsy, cognitive delay, deafness, and blindness in ELBW at a corrected age of 18 months	No difference in the primary composite outcome. Less PDA and severe IVH in the prophylaxis group	Selective use in high-risk infants	In ELBW infants, indomethacin does not improve the rate of survival without neurosensory impairment at 18 months	Indomethacin should not be used as prophylaxis for IVH and PDA as it does not improve survival	Short-term anatomical benefits (lessIVH) don't translate to long-term outcomes Renal risks outweigh neurological benefits in unselected populations Prophylaxis does not lead to neuroprotection
Surrogate-challenging negative	Hydrocortisone for BPD	PREMILOC Trial (2020) [34]	Secondary analysis of RCT (*n* = 295)	Brain abnormality graded by Kidokoro MRI score at term	Higher overall Kidokoro score (5.84 vs. 4.98, *p* = 0.04) but no difference in severe lesions or NDI	Surrogate-clinical discordance	MRI changes did not predict 2-year neurodevelopment	Questions value of routine MRI for glucocorticoid monitoring	Structural MRI changes do not reliably predict neurodevelopmental outcomes Surrogate endpoints (MRI) may not align with clinical outcomes Reinforces need for long-term follow-up in neuroprotection trials
True negative	Hydrocortisone between 7 and 14 days after birth for BPD prevention	SToP-BPD (2019) [35]	RCT (*n* = 372)	Death or BPD at 36 w PMA in mechanically ventilated <30 weeks and/or birth weight of less than 1,250 g	No benefit, similarly, Hydrocortisone at 14–28 days did not prevent BPD [36]	Well-powered null result	Selective use in high-risk infants	Limit hydrocortisone use	Timing (7–14-day delay) does not mitigate adrenal suppression risks BPD pathophysiology may require earlier/later interventions Confirms need for phenotype-specific approaches
Medical reversal	High-dose enteral vitamin A (5,000 IU/kg/day) for BPD prevention	NeoVitaA (2024) [37]	Phase 3 RCT (*n* = 915 ELBW infants)	Moderate/severe BPD or death at 36 weeks PMA	No difference (38% vs. 38%, aOR 0.99 [0.73–1.55]) No change in serum retinol levels Similar AE rates (57% vs. 60%)	Null result despite biologic plausibility	Reverses prior observational/physiological rationale for high-dose therapy	Discontinues experimental high-dose regimens	Even high-dose nutrient supplementation may fail despite strong biologic plausibility Serum retinol levels does not mean clinical efficacy Ends periods of experimentation with high-dose vitamin A regimens
True negative	Lactoferrin to reduce hospital-acquired infections in preterm infants	ELFIN (2019) [38]	RCT (*n* = 2,199)	The aim of this large RCT was to collect data to enhance the validity and applicability of the evidence from previous trials to inform practice	No benefit in using bovine lactoferrin supplement to reduce infections in hospitals. More adverse effects noted in lactoferrin group	Enteral bovine lactoferrin does not reduce late-onset infection in very preterm infants, and current evidence does not support its routine use	No significance despite large sample	Advised not to use lactoferrin to reduce hospital-acquired infections as suggested by several small trials	Largest-ever neonatal nutrition RCT, still null Microbiome complexity limits single-factor solutions Disproves small-study optimism (publication bias)
True negative	Early continuous CPAP vs. surfactant and early intubation in extremely preterm infants	SUPPORT (2010) [39]	Multicenter RCT (*n* = 1,316)	Death or BPD at 36 weeks	No difference in death or BPD between both interventions	True null result showing no difference in outcomes	Well powered study	Early CPAP can be safely considered as an alternative to prophylactic intubation and surfactant in EP infants	Challenged dogma of mandatory early intubation Large trial (*n* = 1,316) showed equipoise Enabled less invasive respiratory management

AE, adverse events; aOR, adjusted odds ratio; APTS, Australian Placental Transfusion Study; BPD, bronchopulmonary dysplasia; CCS, Cognitive Composite Score (Bayley-III); CI, confidence interval; COMET, Cooling for Mild Encephalopathy Trial; CPAP, continuous positive airway pressure; DOLPHIN, Docosahexaenoic acid, choline, and uridine-5-monophosphate supplementation trial; ELBW, extremely low birth weight; EP, extremely preterm; ELFIN, Enteral Lactoferrin in Neonates trial; Epo, erythropoietin; EUNO, European Union Nitric Oxide trial; FGR, fetal growth restriction; HIE, hypoxic-ischemic encephalopathy; HFNC, high-flow nasal cannula; HIP, Hypotension in Preterm infants trial; HIPSTER, High-Flow Nasal Cannula as Primary Support trial; iNO, inhaled nitric oxide; IVH, intraventricular hemorrhage; MRI, magnetic resonance imaging; NDI, neurodevelopmental impairment; NeoVitaA, Neonatal Vitamin A trial; NEC, necrotizing enterocolitis; NEUROSIS, Neonatal European Study of Inhaled Steroids; PDA, patent ductus arteriosus; PENUT, Preterm Erythropoietin Neuroprotection Trial; PH, pulmonary hypertension; PIPS, Probiotics in Preterm infantS trial; PMA, postmenstrual age; POPART, Prophylactic Oropharyngeal Surfactant trial; PREMILOC, PREMature Infants treated with early hydrocortisone: Long-term Outcome study with brain MRI evaluation; RCT, randomized controlled trial; RR, relative risk; SToP-BPD, Steroids To Prevent Bronchopulmonary Dysplasia trial; STRIDER, Sildenafil Therapy in Dismal prognosis Early-onset fetal growth Restriction trial; SUPPORT, Surfactant Positive Airway Pressure and Pulse Oximetry Randomized Trial; TIPP, Trial of Indomethacin Prophylaxis in Preterms.

### True Negative Trials (Definitive No Benefit)

High-quality true negative RCTs that are well-powered but failed to demonstrate a meaningful treatment effect play a critical role in shaping neonatal practice. A good example is the EUNO trial [28], a multicenter European study involving 800 preterm infants that evaluated the use of prophylactic low-dose (5 ppm) inhaled nitric oxide (iNO) for preventing of bronchopulmonary dysplasia (BPD). This trial confirmed no long-term benefits or harms at 2- and 7-year follow-up [40, 41]. Despite its negative outcome, EUNO aligned with prior large studies [42, 43]. It had transformative impacts in resolving debates about prophylactic iNO, shifting the focus to targeted use in pulmonary hypertension, and influencing guidelines, such as those of the American Academy of Pediatrics (AAP) and the European Consensus, to restrict routine iNO use in preterm infants. While disagreements about dosing or subgroups persist, these do not undermine its core findings while leaving room for niche applications.

The use of erythropoietin (Epo) was evaluated in a large RCT. In the PENUT trial [20], extremely preterm (EP) infants who received high-dose Epo had no improvement in survival without neurodevelopmental impairment at 2 years. The PENUT trial findings challenged previous evidence suggesting cognitive benefits of Epo in preterm infants [44]. However, the post hoc analysis of the study findings suggested a decrease in the number of blood transfusions, a clinically significant insight from a negative trial [45].

Another study on Epo’s neuroprotective role in term newborns with hypoxic-ischemic encephalopathy (HIE), the HEAL trial [46] reported no benefit of Epo (relative risk [RR] 1.03, *p* = 0.74) and an increase in adverse events.

The ELFIN trial also exemplifies this category. In this study, over 2,000 infants were randomized to receive bovine lactoferrin for infection prevention. The results challenged previous findings from smaller studies, as there was no reduction in hospital-acquired infections and an increase in adverse events in the treatment group [38].

Table 1 summarizes several examples of negative trials, outlining their principal findings and implications for clinical practice. For example, prophylactic oropharyngeal surfactant was shown to be ineffective [30]. These studies collectively highlight the significance of unfavorable results in the progression of neonatology through the enhancement of evidence-based therapies.

### False-Negative Trials

A false-negative trial arises when a study fails to detect a true treatment effect because of design flaws or inadequate statistical power, leading to an incorrect conclusion of no benefit. In neonatal research, common contributing factors include limited enrollment, inappropriate dosing or timing of therapy, heterogeneity within study populations, inaccurate outcome definitions, and deviations from trial protocols or crossover between treatment arms.

On the topic of probiotic prophylaxis, PiPS trial is the largest trial to date, enrolling 1,315 participants and evaluating *Bifidobacterium breve* BBG-001 for prophylaxis in extremely low birth weight (ELBW) infants [22]. While the study confirmed the short-term safety of this probiotic, it found no clinical advantage from using this particular strain, particularly in reducing necrotizing enterocolitis (NEC) or mortality.

In contrast, several trials and meta-analyses suggest that multi-strain probiotic regimens, such as those combining *Lactobacillus with Bifidobacterium*, may be beneficial [47]. Within the PiPS trial, a secondary evaluation of colonization data (*n* = 1,186) showed non-significant trends toward lower rates of NEC (odds ratio [OR] 0.68), sepsis (OR 0.88), and mortality (OR 0.68) among infants who achieved successful colonization.

An important limitation was the unexpectedly high rate of colonization in the placebo group (37% at 2 weeks and 49% by 36 weeks PMA), indicating that future studies may need to consider cluster-randomized approaches [22]. Initially, colonization was significantly reduced with exclusive breast milk (OR 0.52), compared with formula use (OR 2.21); however, these associations were not significant after adjustment in multivariable models.

The ProPrems trial [48], which evaluated a different probiotic combination (*Bifidobacterium infantis*, *Streptococcus thermophilus,* and *Bifidobacterium lactis*), found no benefit for sepsis or mortality but did demonstrate a reduction in NEC (4.4%–2.0%; RR 0.46). Although PiPS was a high-quality study, its negative result may represent a false negative attributable to the use of a single strain. Current evidence suggests that multi-strain probiotics may be more effective [49, 50], which supports their continued use by many neonatologists, despite the discouraging findings of the PiPS trial.

The APTS trial [17], a large multicenter RCT in preterm infants <30 weeks, compared delayed cord clamping (DCC; ≥60 s) with immediate clamping (≤10 s). The trial failed to show a significant reduction in the primary outcome of death or major morbidity at 36 weeks. However, it showed favorable secondary outcomes, such as fewer transfusions and higher hemoglobin levels, without any added risks, including hyperbilirubinemia or intraventricular hemorrhage (IVH).

Although 15% of infants in the DCC group did not receive the intervention as assigned, mainly due to clinical instability or provider choice, the APTS trial still shaped international recommendations. Both the World Health Organization (WHO) and the AAP now endorse DCC for 30–60 s, based on its demonstrated safety. While the trial’s primary composite outcome was negative, unadjusted mortality appeared lower with DCC (6.4% vs. 9.0%, RR 0.69), though this effect lost significance after adjustment. The study was not powered to detect mortality differences; however, subsequent meta-analyses [51] pooling 1,268 infants across 17 trials found that placental transfusion reduces preterm mortality by roughly 30% (RR 0.71), even when APTS data were excluded (RR 0.56). Early termination can compromise study power and lead to false-negative conclusions, as illustrated later in the HIPSTER trial [27].

### Equivocal/Inconclusive Trials

Equivocal trials are studies in which the results neither confirm nor rule out a treatment benefit. Such outcomes often arise from wide confidence intervals, premature trial termination, or high rates of dropout.

The HIP trial [26] illustrates the challenges of futility designs, as it was stopped early after enrolling only 58 of the 830 planned EP infants in a comparison of dopamine versus placebo (restrictive approach) for hypotension. No significant difference was seen in survival without brain injury (62% vs. 69%, *p* = 0.58), but the trial’s extreme underpowering (7.7% of target enrollment) rendered results uninterpretable. An important finding was that the restrictive approach reduced the need for additional treatments (38% vs. 66%, *p* = 0.038). However, the small sample size prevented firm conclusions about safety or efficacy, underscoring both the clinical uncertainty and broader structural barriers in neonatal cardiovascular research.

The COMET pilot RCT [29] evaluated whole-body hypothermia for infants with mild HIE and found no improvement in cerebral magnetic resonance imaging (MRI) biomarkers. Baseline illness severity was higher in the cooled group. While the results were statistically negative, they remain clinically inconclusive and highlight the need for larger, definitive trials in this understudied population.

A randomized trial comparing sustained versus standard lung inflation in EP infants (23–26 weeks’ gestation; SAIL trial) [52] was terminated early and showed no significant difference in the combined outcome of BPD or death (63.7% vs. 59.2%, *p* = 0.29). Interpretation was limited by possible unmeasured variation in how the intervention was applied, despite standardized training, and by insufficient power, leaving the study unable to draw firm conclusions, even though outcomes did not differ across regions [53].

### Harmful Interventions Detected by “Negative” Trials

Not all negative trials demonstrate futility alone – some reveal unanticipated harm, leading to critical practice changes. In neonatology, several high-profile RCTs have challenged long-standing interventions by identifying either direct harm or failure to improve meaningful outcomes, especially in vulnerable preterm populations.

The Doyle et al. [54] meta-analysis of 26 RCTs (*n* = 3,700) re-evaluated early dexamethasone for BPD prevention. Although widely used for decades, the findings revealed a significant increase in cerebral palsy when dexamethasone was administered to infants with a <30% baseline BPD risk. Benefits were only seen in high-risk subgroups (>70% risk), making the case for individualized use rather than blanket administration – ultimately reversing indiscriminate steroid use in low-risk infants [54].

The NEUROSIS trial [23] examined early inhaled budesonide and demonstrated a reduction in BPD, aligning with biological plausibility and earlier meta-analyses [55]. However, it raised concern with a non-significant yet worrying trend toward higher mortality (16.9% vs. 13.6%; RR 1.24, 95% CI 0.91–1.69; *p* = 0.17). Although the cause of death was not clearly linked to infection or any specific mechanism [56], and some experts argued the trend might reflect chance or site-level variation, the trial was interpreted by many as potentially harmful. This ambiguity shifted clinical enthusiasm and illustrated how unclear mortality signals can override efficacy – even when the primary endpoint is positive.

The SUPPORT [46] and BOOST-II [19] trials investigated optimal oxygen saturation targets in preterm infants. While aimed at reducing retinopathy of prematurity (ROP), both trials found that lower oxygen targets (85–89%) were associated with higher mortality – 19.9% vs. 16.2% in SUPPORT (RR 1.27, 95% CI 1.01–1.60; *p* = 0.04), and 23.1% vs. 15.9% in BOOST-II (RR 1.45, 95% CI 1.15–1.84; *p* = 0.002). BOOST-II also showed a rise in NEC with lower oxygen (10.4% vs. 8.0%; RR 1.31, 95% CI 1.02–1.68; *p* = 0.04). While high oxygen levels increased ROP, these trials revealed a harmful trade-off that forced the neonatal community to adopt a safer middle range (90–95%) for oxygen saturation targets [57].

In the NICHD Neonatal Research Network trial [58], aggressive phototherapy was studied in 1,974 ELBW infants across 16 centers. While aggressive phototherapy reduced neurodevelopmental impairment in larger ELBW infants, it unexpectedly increased mortality by 5–8% among ventilated infants ≤750 g. Bayesian analyses estimated a >99% probability of harm in this subgroup, challenging the assumption of universal phototherapy safety and raising concerns about oxidative or hemodynamic stress. This trial emphasized that beneficial therapies in one subgroup can be dangerous in others.

The HEAL trial [46], which evaluated Epo as a neuroprotective drug in term infants with HIE, found no improvement in death or neurodevelopmental impairment compared to placebo. Despite promising preclinical data and earlier pilot studies, the results showed futility in using Epo as an adjunct to hypothermia, prompting reconsideration of its clinical use.

Similarly, the PREMOD2 trial [59], which compared umbilical cord milking (UCM) to DCC in preterm infants <32 weeks, was terminated early due to a concerning rise in severe IVH in the UCM group. Though no difference was seen in the primary outcome of death or severe IVH, the early harm signal highlighted the risks of UCM and reinforced current recommendations favoring DCC for hemodynamic stability and neuroprotection.

### Trials with Opposite or Paradoxical Effects

Several landmark neonatal trials have demonstrated interventions causing effects directly opposing their intended purpose. The Dutch STRIDER trial (2022) evaluated antenatal sildenafil for growth-restricted fetuses but found no reduction in perinatal mortality/morbidity. Paradoxically, neonates exposed to sildenafil developed higher rates of pulmonary hypertension (18.8% vs. 5.1% in controls (placebo group); RR 3.67, 95% CI 1.28–10.51, *p* = 0.02), a strikingly opposite effect since sildenafil is clinically used as a pulmonary vasodilator to treat this exact condition. This reversal likely occurred due to rebound vasoconstriction after birth when fetal drug exposure ceased, leading to immediate discontinuation of this experimental therapy [32].

The SAIL trial’s sustained inflations during preterm resuscitation showed similarly counterintuitive outcomes [52]. Despite theoretical benefits, the intervention was linked to excess early deaths (11/215 intervention vs 1/211 controls), particularly at 23–24 weeks’ gestation, forcing abandonment of the technique. Earlier meta-analyses (*n* = 941) had already shown no mortality benefit of sustained inflations (RR 1.01, 95% CI 0.67–1.51) [60]), with no differences in most secondary outcomes, but SAIL revealed unexpected vulnerability in EP infants.

In line with concenrs raised by the earlier patent ductus arteriosus (PDA)-Tolerate Trial [61], which showed no benefit to routine pharmacological ductal closure at the end of the first week, susbsequent trials employing early or targeted ibuprofen treatment (BeNeDuctus and Baby-OSCAR, *n* = 926 combined) similarly failed to demonstrate improved outcomes comapred with conservative management. BeNeDuctus showed increased BPD (50.9% vs. 33.3%), while Baby-OSCAR revealed concerning mortality trends (13.6% vs. 10.3%), proving that pharmacologic PDA closure does not necessarily improve outcomes [24, 62]. Moreover, the recently published 2-year neurodevelopmental follow-up of the Baby-OSCAR trial demonstrated no benefit of early PDA treatment [25].

The NEOPAIN trial (*n* = 898) revealed another paradox: preemptive morphine in ventilated preterm failed to reduce death/severe brain injury while increasing harm in subgroups. Neonates receiving only protocol morphine (no rescue doses) developed higher rates of severe IVH (9% vs. 3%, *p* = 0.02) and composite adverse outcomes (24% vs. 15%, *p* = 0.03), showing that routine opioid use in ventilated preterm infants may counteract its analgesic benefits with neurological risks [63].

### Trials Challenging Surrogate Outcomes

Several studies have demonstrated significant limitations when using surrogate markers to predict clinical benefits in neonatal medicine. These studies highlight important gaps between measurable biological effects and meaningful patient outcomes.

The experience with superoxide dismutase illustrates this clearly: although preclinical and early phase studies demonstrated reductions in oxidative stress markers [64], subsequent clinical trials failed to show improvements in severe ROP or mortality [65]. This was despite a strong mechanistic rationale targeting free radical-mediated injury, a known driver of ROP pathogenesis.

A second landmark example is the TIPP trial (2001), where indomethacin effectively reduced PDA and IVH, both widely used surrogate endpoints, but did not improve survival without disability [33]. This conclusion held even after post hoc adjustment for antenatal steroid exposure (*p* = 0.15 for interaction) [66].

Neuroimaging studies have revealed similar limitations. The PREMILOC trial’s MRI sub-study [34] found hydrocortisone associated with higher overall Kidokoro scores (5.84 vs. 4.98, *p* = 0.04), yet this difference showed no correlation with clinically significant brain lesions (Kidokoro ≥6, *p* = 0.38) or subsequent neurodevelopmental impairment [67]. This dissociation suggests imaging biomarkers may reflect normal maturational variations rather than pathological changes.

The NEOPAIN trial [63] further demonstrated that while morphine achieved adequate analgesia in preterm infants, it had no impact on mortality or long-term outcome. Conversely, it was associated with increased neurological risks.

### Trials Leading to Medical Reversals

Several high-quality neonatal trials have led to medical reversals, where once-accepted interventions were later disproven despite biological plausibility or early encouraging results. The NeoVitaA trial [37] overturned the longstanding hypothesis that high-dose enteral vitamin A (5,000 IU/kg/day) reduces BPD in ELBW infants. Despite vitamin A’s known role in lung development, the trial showed no benefit in the composite outcome of moderate/severe BPD or death (38% in both groups; adjusted OR 0.99, 95% CI 0.73–1.55), and no increase in serum retinol levels indicating poor absorption and challenging prior assumptions. A previous meta-analysis had also shown inconsistent benefits of vitamin A supplementation in preterm infants [68], reinforcing these findings.

A pivotal systematic review by Saugstad et al. [69] including 10 randomized and quasi-randomized trials (*n* = 2133) demonstrated that using 21% oxygen (room air) for neonatal resuscitation significantly reduced neonatal mortality by 31% (RR 0.69, 95% CI 0.54–0.88) compared to 100% oxygen, with a trend toward lower rates of severe HIE. These results reversed a century-old global practice of using pure oxygen at birth and led to a landmark change in the 2010 ILCOR guidelines, making room air the standard of care.

The HIPSTER trial [27] was another example of a reversal. A study compared high-flow nasal cannula (HFNC) with continuous positive ariway pressure (CPAP) in preterm infants and found that HFNC was not non-inferior; treatment failure occurred in 25.5% of infants treated with HFNC vs. 13.3% of those treated with CPAP (*p* < 0.001). Although HFNC was considered more comfortable and easier to use, the evidence showed CPAP’s clear superiority, highlighting the risks of prematurely adopting convenient alternatives without robust data.

In the PREMOD2 trial [59], early termination occurred after preliminary results showed higher rates of severe IVH with UCM compared to DCC in infants <32 weeks’ gestation. While previous studies supported hemodynamic and neurodevelopmental benefits of DCC, PREMOD2 indicated that UCM might be a risky practice for preterm infants. Due to the early termination of the study, definitive conclusions were limited, and significant safety concerns were raised. Additional notable examples include the SAIL trial [52], where sustained inflation, despite physiological rationale, led to increased mortality without reducing BPD, halting its clinical use. The NEOPAIN trial showed that routine opioid use for mechanically ventilated preterm infants did not improve neurodevelopment and was associated with adverse effects [63]. Furthermore, the TIPP trials [33, 66] reversed the routine use of early prophylactic indomethacin as they failed to show an improvement in survival without neurological risk despite reducing rates of PDA and IVH.

## Why Negative Results Matter in Neonatology

Negative studies have shaped neonatology by challenging assumptions, reducing redundant research, and redirecting resources. For example, the COMET study identified unexpectedly greater cerebral MR metabolic abnormalities in mild HIE neonates who underwent whole-body hypothermia, prompting new research questions about the biological siginficance of mild HIE and the role of late colling [29]. Similarly, after the EUNO tiral demonstrated no benefit of prophylactic iNO in preterm infants, the NIH discontinued further iNO studies after the EUNO trial, reallocating resources to more promising areas in neonatal practice research [28].

Table 2 summarizes the key rationales for publishing negative results in neonatal research, supported by illustrative examples from pivotal clinical trials. These examples highlight how null or unfavorable outcomes help generate new hypotheses, prevent harm, strengthen meta-analyses, and uphold ethical and regulatory standards.

**Table 2.** Value and rationales for publishing negative results in neonatal research: key justifications with illustrative examples

No.	Significance	Rationale	Discussion	Real neonatal example
1	Hypothesis-generating	Uncover unexpected findings to guide future studies	Reveal critical knowledge gaps and challenge assumptions about extending therapies from high-risk to borderline populations	The COMET trial [29], while finding no benefit of hypothermia for mild HIE on MR biomarkers (NAA levels: 10.98 vs. 8.36/9.02 mmol/kg), generated two key hypotheses: (1) the paradoxical NAA reduction in cooled infants may reflect altered metabolic adaptation rather than injury, and (2) baseline illness severity differences (45% intubation in hypothermia vs. 9% normothermia groups) suggest mild HIE is heterogenous and may require risk-stratified approaches. This underscores how negative pilot trials can refine research questions
2	Avoid duplication of research efforts	Prevents repeating unsuccessful studies	Publishing negative studies ensures visibility of previous negative outcomes, preventing unnecessary trials that waste resources and involve vulnerable neonates	The 2011 NIH Consensus Statement [70], issued after the EUNO trial [28] results, made key recommendations: (1) explicitly advised against routine use of iNO in preterm infants <34 weeks, stating “there is no evidence supporting its clinical use,” and (2) redirected NIH funding toward alternative BPD research priorities
3	Prevent harm from ineffective/dangerous interventions	Protects neonates from harmful or ineffective treatments	Negative results are vital to identifying interventions with unfavorable risk-benefit ratios, thus safeguarding neonates from unnecessary and harmful therapies	The Canadian STRIDER trial [71] was halted in 2018 after the Dutch STRIDER trial signaled the potential for harm of increased neonatal pulmonary hypertension with maternal sildenafil, preventing unsafe adoption despite mechanistic plausibility [32]
				SUPPORT [72] and BOOST-II [19] trials demonstrated increased mortality with higher oxygen saturation targets (91–95%) compared to lower targets (85–89%)
4	Strengthen meta-analyses and evidence synthesis	Ensures balanced and accurate evidence synthesis	Including negative results in meta-analyses reduces publication bias, correcting inflated effect sizes and leading to more accurate clinical recommendations	The 2020 Pammi meta-analysis [73], which incorporated the large ELFIN trial (*n* = 2203) [38], reversed the 2017 conclusion that lactoferrin reduced late-onset sepsis (RR 0.80 vs. prior 0.59) and eliminated the apparent benefit for NEC (RR 1.10 vs. prior 0.40), demonstrating how definitive trials can correct overoptimistic estimates from smaller studies
5	Uphold ethical obligations	Honors participants’ contributions by transparently reporting all outcomes	The Council for International Organizations of Medical Sciences (CIOMS) International Ethical Guidelines for Health-Related Research Involving Humans (2016) [74] explicitly states: “Negative and inconclusive as well as positive results of all studies should be published or otherwise be made publicly available” (Guideline 24). This ethical imperative ensures research integrity, honors participant contributions, and prevents publication bias that could mislead clinical practice	The PENUT [20] and ELFIN [38] trials’ publication of null results adhered to CIOMS Guideline 24 [74] by transparently reporting inefficacy, ensuring that the contributions of close to 2,000 study participants advanced neonatal care despite negative outcomes. This fulfilled ethical obligations to research participants while preventing unnecessary duplication of research efforts
6	Identify beneficial subgroups	Reveals subgroup-specific benefits despite overall negative results	Negative studies can identify beneficial effects within subgroups, supporting precision medicine and targeted intervention strategies in neonatology	In the PREMILOC trial’s prespecified subgroup analysis, hydrocortisone significantly reduced moderate-to-severe NDI in infants born at 24–25 weeks’ gestation (2% vs. 18%; adjusted risk difference 16%, 95% CI −28% to −5.0%, *p* = 0.02) [67, 75]
7	Improve methodological rigor	Helps refine clinical trial methods and design for improved future research quality	Negative outcomes highlight methodological flaws, prompting better-designed future trials with improved power, sample sizes, and outcome assessments	TOBY trial (2009) [76] improved the robustness of its study by expanding its sample size to 325 infants beyond its initial target based on lessons from prior trials (CoolCap [2005, *n* = 234] [77] and NICHD [2005, *n* = 239] [78], which were underpowered due to overestimated poor outcome rates in controls (CoolCap: 70%; NICHD: 50%)
8	Demonstrate cost-effectiveness	Prevents adoption of costly, ineffective treatments, optimizing resource use	Publishing negative studies avoids unnecessary expenditures by clarifying ineffective treatments, guiding resource-efficient practices in neonatal care	Negative trials have driven cost-effective neonatal care by exposing futile practices: abandoning prophylactic probiotics in ≥32-week infants when NEC risk is <6.5% (incremental cost per QALY exceeded willingness-to-pay thresholds), [79] discontinuing routine morphine infusions in ventilated preterms (NEOPAIN trial) [63], avoiding universal surfactant administration (CURPAP trial) [80], and restricting inhaled iNO in preterm RDS [41–43], particularly prophylactic use of iNO which has poor cost-effectiveness profile [81]
9	Correct publication bias	Ensures comprehensive evidence base for accurate clinical decision-making	Negative study publication counteracts bias towards positive results, ensuring that clinical decisions are based on a truthful representation of intervention effectiveness	CURPAP and SUPPORT trials of prophylactic surfactant showed no advantage over CPAP or selective use, correcting bias from earlier positive reports [39, 80]
10	Enhance clinical education and decision-making	Educates clinicians on true effectiveness, improving clinical judgment and guidelines	Negative results teach clinicians critical appraisal skills, preventing misconceptions and overly optimistic expectations of interventions	The PREMILOC trial follow-up showed no neurodevelopmental benefit from early low-dose hydrocortisone in 26-27 weeks, despite promising short-term outcomes. This highlights the importance of considering long-term effects in clinical decision-making [67, 75]
11	Inform regulatory decisions	Provides necessary comprehensive evidence for informed regulatory approvals and practice guidelines	Regulators rely on negative trial data to prevent approval of ineffective or harmful therapies, ensuring patient safety through evidence-informed decisions	Negative trials of iNO in preterm infants prompted regulatory statements against routine or prophylactic use [70, 81–84]

BPD, bronchopulmonary dysplasia; CI, confidence interval; CIOMS, Council for International Organizations of Medical Sciences; CPAP, continuous positive airway pressure; CURPAP, curative CPAP vs. prophylactic surfactant trial; ELFIN, Enteral Lactoferrin in Neonates trial; GA, gestational age; HIE, hypoxic-ischemic encephalopathy; iNO, inhaled nitric oxide; MR, magnetic resonance; NAA, N-acetylaspartate; NDI, neurodevelopmental impairment; NEC, necrotizing enterocolitis; NEJM, New England Journal of Medicine; NIH, National Institutes of Health; NNT, number needed to treat; NR, not reported; PREMILOC, Early Low-Dose Hydrocortisone to Improve Survival Without BPD in Extremely Preterm Infants trial; QALY, quality-adjusted life year; RCT, randomized controlled trial; RR, relative risk; STRIDER, Sildenafil TheRapy In Dismal prognosis Early-onset intrauterine growth Restriction trial; SUPPORT, Surfactant Positive Airway Pressure and Pulse Oximetry Randomized Trial; TOBY, Total Body Hypothermia trial.

Negative trials protect infants from harmful treatments, such as sildenafil in the STRIDER trials [32, 71], and prevent the adoption of ineffective therapies. Their inclusion in meta-analyses corrects biased evidence, as demonstrated by the ELFIN’s null result on lactoferrin [38]. Negative findings also improve trial methodology, as seen in the DOLPHIN and TOBY trials [31, 76], and fulfill ethical obligations through transparent reporting [74]. They further support precision medicine by identifying responsive subgroups [75], guide cost-effective care by eliminating futile interventions [63, 79–81], and inform regulatory policies with accurate risk-benefit profiles [70, 82–84].

As neonatology continue to evolve, the field depends on a steady foundation of rigorous evidence that refutes wildely held-assumptions. There is an urgent need for robust, well-designed clinical trials in neonatology, particularly in core fields such as pharmacology, respiratory management, cardiovascular support, nutrition, and neuroprotection [8, 85]. Other important areas include infection control, long-term neurodevelopment, family-centered care, translational and genomic research, and implementation science. Importantly, the results of such trials should be shared and applied, regardless of whether the outcomes are positive, negative, or inconclusive [8]. From strengthening research integrity to improving clinical education, negative neonatal trials turn setbacks into building blocks for evidence-based practice [75, 80].

## Limitations

This review has limitations. Unlike systematic or scoping reviews, our methodology was not based on predefined eligibility criteria or comprehensive database searching. Instead, we purposively selected interventional trials that illustrate the role of negative results in neonatal care research. As such, this review may not capture all relevant studies, and selection bias cannot be excluded. Nevertheless, our intent was not to provide exhaustive coverage, but to highlight conceptual and practical lessons from representative trials.

## Conclusion and Future Directions

Neonatal research trials with negative results are considered essential milestones in advancing safer and more effective neonatal care. Their value lies in preventing harm, refining therapies, and shaping research priorities. Neonatologists and researchers must address publication bias by committing to full transparency, starting with trial registration, including open data sharing, and publishing all results, regardless of the trial outcome.

NICU practices and future research in neonatology should adopt care bundle approaches by combining several evidence-based interventions and targeting shared outcomes. Furthermore, enhancing multicenter collaborations will improve statistical power, diversity, and generalizability of findings.

In conclusion, negative results in neonatal clinical trials, when derived from reliable and adequately powered studies, are invaluable for evidence-based practice. They prevent the continuation of ineffective or harmful interventions, refine research priorities, and guide clinical care. The key message is that reliability, through robust design, transparent registration, and clinically meaningful outcomes, is more important than whether a result is positive or negative.

## Acknowledgments

We thank Sereen Gad for her careful review of the manuscript’s references, which enhanced its accuracy.

## Conflict of Interest Statement

The authors declare no conflict of interest.

## Funding Sources

Open access funding provided by the Qatar National Library. This research did not receive any other grants from funding agencies in the public, commercial, or not-for-profit sectors.

## Author Contributions

A.G. conceptualized the review, conducted literature searches, and wrote the first draft; A.Y.N. contributed to writing and critical review; L.K.A.-Q. conducted literature searches and contributed to writing and critical review; L.A. participated in writing, conducted literature searches, and critical review. All authors reviewed and approved the final manuscript.
